# Association between obesity indexes and chronic kidney disease risk: a double-cohort prospective study in the Binhai and UK Biobank

**DOI:** 10.3389/fendo.2025.1566011

**Published:** 2025-06-27

**Authors:** Suhua Gao, Yixi Liu, Hongyan Liu, Yao Lin, Pufei Bai, Fang Hou, Shan Lu, Saijun Zhou, Haizhen Sun, Guangyang Ma, Hao Liu, Mianzhi Zhang, Zhuang Cui, Pei Yu

**Affiliations:** ^1^ National Health Commission (NHC) Key Laboratory of Hormones and Development, Chu Hsien-I Memorial Hospital and Tianjin Institute of Endocrinology, Tianjin Medical University, Tianjin, China; ^2^ Tianjin Key Laboratory of Metabolic Diseases, Tianjin Medical University, Tianjin, China; ^3^ Department of Nephrology, Xi’an Jiao Tong University-affiliated Honghui Hospital, Xi’an, China; ^4^ Community Health Service Center, Tianjin, China; ^5^ Department of Radiology, Chu Hsien-I Memorial Hospital and Tianjin Institute of Endocrinology, Tianjin Medical University, Tianjin, China; ^6^ Department of Nephrology, Dongfang Hospital of Beijing University of Chinese Medicine, Beijing, China; ^7^ Department of Epidemiology and Health Statistics, Tianjin Medical University, Tianjin, China

**Keywords:** obesity, chronic kidney disease, Chinese visceral adiposity index, obesity indexes, double-cohort study

## Abstract

**Introduction:**

The prevalence of overweight and obesity has increased worldwide, leading to growing concern regarding the impact of visceral adipose deposition on renal function. The aim of this study was to evaluate the predictive value of 10 obesity indexes for the risk of chronic kidney disease (CKD) in both Chinese populations and Western.

**Methods:**

The Tianjin Chronic Kidney Disease Study (Binhai, primary cohort) included 126,109 participants, while 358,918 adults from the U.K. Biobank (UKB, replication cohort) were included. Cox proportional hazard and restricted cubic spline models were used to assess the relationships between obesity indexes and the risk of CKD.

**Results:**

During a median follow-up of 35 months in the Binhai cohort, 14,435 CKD cases were identified, while 358,918 CKD cases were observed in the U.K. Biobank cohort during 161 months of follow-up. The risk of CKD increased with increasing quartile levels of the Chinese Visceral Adiposity Index (CVAI) (P for trend < 0.001). CVAI was associated with increased CKD risk (hazard ratio in comparing the highest to the lowest quintile = 1.22 [95% CI 1.16-1.30]) and its predictive ability was the highest among the 10 obesity indexes, with an AUC value of 0.588 (0.581-0.594) in the female subgroup of the Binhai cohort. All of the obesity indexes were negatively correlated with estimated glomerular filtration rate (eGFR).

**Discussion:**

Findings from two large prospective cohort studies support the notion that obesity indexes, particularly CVAI, are significantly associated with the risk of CKD across diverse ethnic groups.

## Graphical Abstract

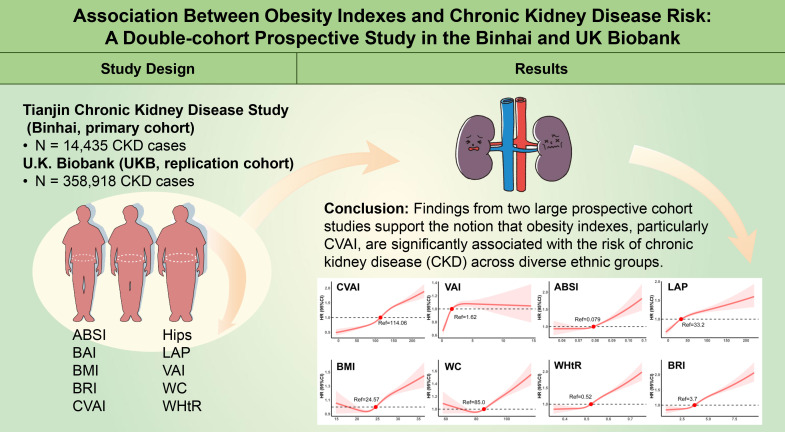

## Introduction

Based on the findings from the Global Burden of Disease Study, the global prevalence of chronic kidney disease (CKD) reached 697.5 million cases in 2017, with China alone contributing 132 million CKD patients, marking a significant 29.3% increase since 1990 (1). The prevalence of CKD is reported to be 23% among individuals with obesity (2).

Among the several clinical evaluation indicators, the body mass index (BMI) remains the most widely used and recognized measure for identifying obesity (3). However, it is important to note that obesity, especially visceral obesity, is strongly associated with the prevalence of CKD (4). Thus, the assessment of visceral obesity is critical for the development of strategies aimed at preventing CKD.

Waist circumference (WC), hip circumference, and waist-to-hip ratio (WHtR) are commonly used measures to differentiate between peripheral and central obesity. Furthermore, body fat content and visceral fat area, as derived from anthropometric measures, may also be applied to the evaluation of obesity (5). Computed Tomography (CT) and Magnetic Resonance Imaging (MRI) are both commonly used to assess visceral obesity. However, their high cost restricts its application in routine clinical practice and large-scale population screening. In 2016, the Chinese Visceral Adiposity Index (CVAI), validated through CT, was established as a clinical index specifically intended to predict visceral fat function in Asian populations (6). A study indicated that CVAI outperforms both body mass index (BMI) and waist circumference (WC) in effectively predicting the risk of prediabetes and diabetes in Chinese adults (7). Several studies have shown that CVAI is associated with the development of diabetes and offers higher predictive validity compared to other indicators (8–10). Among the indexes of neck circumference, WHtR, lipid accumulation product (LAP), VAI, and CVAI, it has been found that CVAI exhibits the strongest association with cardiovascular and cerebrovascular diseases, as well as diabetic kidney disease (11). However, existing evidence regarding the relationship between CVAI and CKD remains limited, with a lack of comprehensive data derived from large-scale prospective cohort studies.

To address the gaps in understanding the relationship between obesity indexes and CKD, we conducted research to: 1) investigate the influence of several obesity indexes, including ABSI, BAI, BMI, BRI, CVAI, Hips, LAP, VAI, WC and WHtR, on the incidence of CKD using data from the Binhai and U.K. Biobank cohorts; 2) evaluate the differences and consistency in the associations between these indexes and CKD risk across the two cohorts; and 3) explore how these indexes, in combination with established CKD-related indicators, may enhance predictive models for CKD incidence.

In this research, we prospectively investigated the relationship between obesity indexes and the risk of CKD using data from two large-scale cohorts: 126,109 Chinese community-based participants and 358,918 patients from a diverse, multi-ethnic population. This double-cohort analysis provides novel insights into the role of obesity indexes in CKD risk, marking the first comprehensive study to compare and contrast these correlations across diverse populations.

## Materials and methods

### Study design and participants

**Figure 1:**
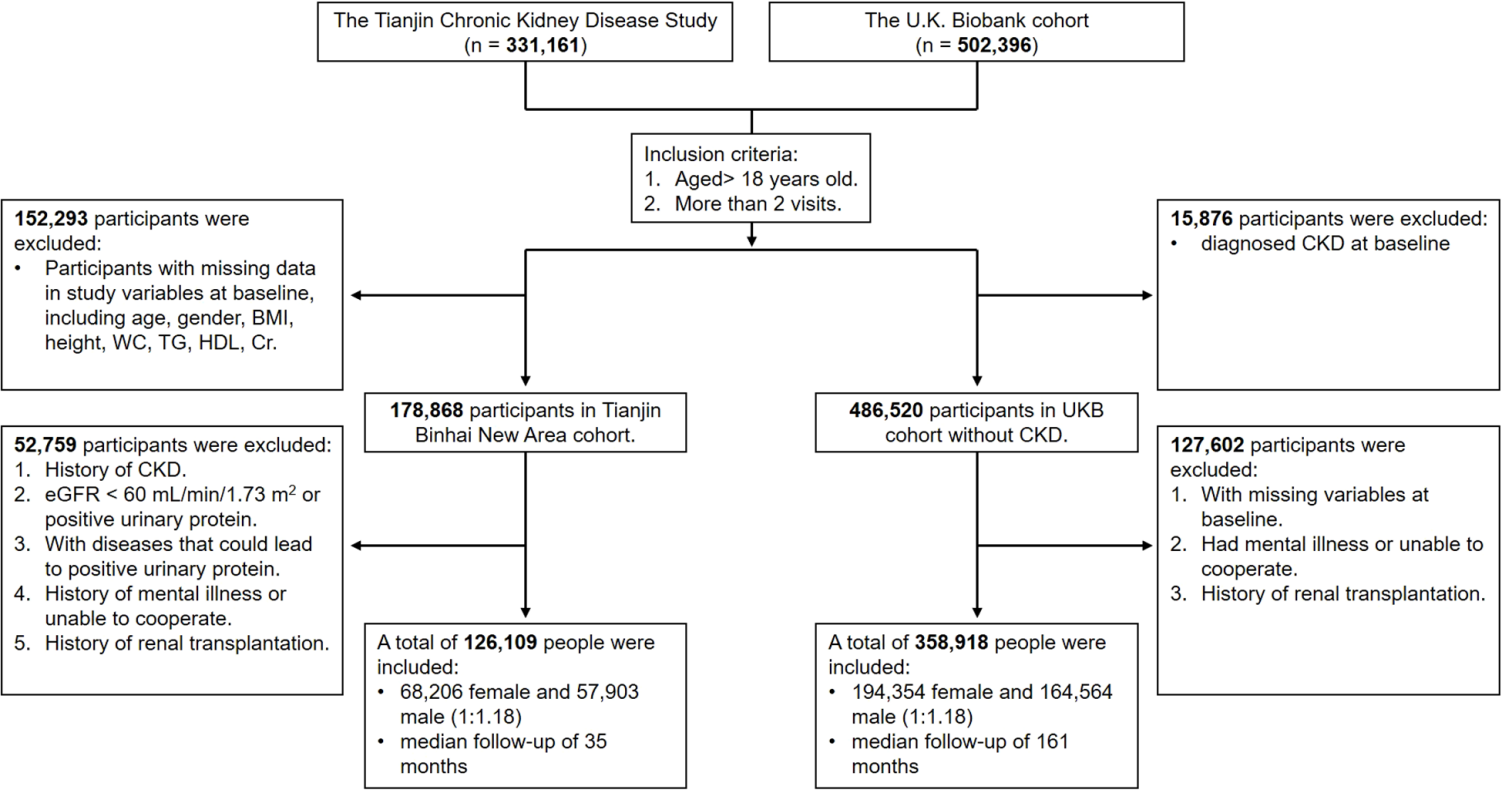
Study flow chart.

We included the Tianjin Chronic Kidney Disease Study (Binhai) as the primary cohort and U.K. Biobank (UKB) as the replication cohort. The participants in this study were adults over 18 years old in the Binhai New Area, Tianjin, China (12). This study is a prospective cohort study initiated in January 2013, designed to investigate the underlying patterns in the occurrence and progression of chronic diseases in the elderly population. The adults who received regular physical examinations were followed up for 3 years from 2018 to 2021. The median interval of the visits for the participants is 37 months. Inclusion criteria: 1. Aged > 18 years old; 2. More than 2 visits during the 3 years, including the baseline visit. Exclusion criteria: 1. History of CKD; 2. eGFR < 60 mL/min/1.73 m^2^ or positive urinary protein (ACR ≥ 30 mg/g or urinary protein ≥ 1+); 3. With diseases that could lead to positive urinary protein; 4. With missing variables at baseline; 5. Had mental illness or unable to cooperate; 6. History of renal transplantation. According to the inclusion and exclusion criteria, a total of 126,109 participants (68,206 female and 57,903 male) were finally enrolled in the Binhai cohort (**Figure 1**). In the U.K. Biobank cohort, we included adult participants aged 18 and above, with baseline data collected from 2006 to 2010 and follow-up until September 2022. According to the inclusion and exclusion criteria, a total of 358,918 participants (194,354 female and 164,564 male) were finally enrolled in the U.K. Biobank cohort (**Figure 1**).

### Diagnostic criteria of CKD

According to the 2020 Kidney Disease: Improving Global Outcomes clinical practice guidelines for the management of CKD, the definition is abnormal renal structure or function for more than 3 months (13). Any of the following indicators last for more than 3 months can be used to diagnose CKD: 1. The signs of renal injury were as follows: a. positive urinary protein (ACR ≥ 30 mg/g or urinary protein ≥ 1+); b. abnormalities in urinary sediment as markers of kidney damage; c. pathological abnormalities. 2. eGFR < 60 ml/min/1.73 m^2^ (eGFR stage: G3a~G5 stage). The eGFR was calculated by the CKD-EPI formula (14). In the U.K. Biobank cohort, CKD was defined using ICD10 codes from hospital inpatient records (N03.*, N06.*, N08.*, N11.*, N12.*, N13.*,N14.*, N15.*, N16.*, N18.*, N19.*, N20.*, N21.*, UKB field 41270: https://biobank.ndph.ox.ac.uk/ukb/field.cgi?id=41270) (15, 16).

### Data collection

The Binhai cohort data included demographic characteristics (age and sex), physical measurements (height, weight, BMI, WC, blood pressure), lifestyle (smoking status, drinking frequency, dietary habits, and physical activity), medication history, history of diseases (type 2 diabetes, hypertension, stroke), and laboratory examination. Physical activity frequency was categorized as never, 1 time per week, 1–7 times per week, and >7 times per week. Dietary habits were categorized as balanced meat consumption and imbalanced meat consumption (vegetarian-based, meat-based, heavy salt, heavy oil, and heavy sugar). Smoking status: current, never, and quit. Drinking frequency: never, 1, 1-7, and >7 times per week. Laboratory tests included HB, FBG, AST, ALT, TB, Cr, BUN, TC, TG, LDL-c, HDL-c.

The calculation of obesity indexes was based on the formulas provided in **Supplementary Table S1**. Experienced physicians and nurses from community and medical examination centers completed the data collection and entered the data into the Tianjin Community Health Service Center for management. The original data can be accessed and downloaded by logging into the system. The U.K. Biobank cohort data were collected through the UKB database, as described in our previous study (17–19).

### Statistical analyses

After conducting normality tests, continuous variables with normal distribution were described by mean ± standard deviation (mean ± SD). An independent samples t-test and one-way analysis of variance (ANOVA) were employed to compare between two groups and multiple groups. Non-normally distributed data were expressed as median (25th-75th percentile), and the standard Wilcoxon rank sum test was used for comparison. Categorical variables were presented as frequency (N, %) and analyzed using the Chi-square (χ²) test. The association between obesity indexes and eGFR was examined using Spearman’s rank correlation analysis, and the Cochran-Armitage (CA) trend test was employed to determine whether a trend existed among categorical variables.

In addition, a Cox proportional hazards regression model was employed to assess the association between obesity indexes and the risk of CKD, with hazard ratio (HR) and corresponding 95% confidence intervals (95% CIs) calculated to quantify the strength and precision of the associations. Specifically, we considered the following series of models: 1) in Model 2, adjustments were made for age and gender; 2) in Model 3, a more comprehensive adjustment was applied, accounting for age, gender, systolic and diastolic blood pressure, lipid profiles (including LDL-c, HDL-c, TC, TG), Cr, eGFR, smoking status, drinking frequency, dietary habits, physical activity frequency, and the presence of comorbid conditions such as diabetes, hypertension, and a history of stroke.

In the trend analysis, obesity indexes were categorized into quartiles, with the median value of each quartile incorporated into the regression model.

To explore the nonlinear relationship between obesity indexes and the risk of CKD, we employed a restricted cubic spline (RCS) regression model to analyze the dose-response association. The receiver operating characteristic (ROC) curve was used to calculate the area under the curve (AUC) and 95% confidence interval (CI) predicted by obesity-related indicators for CKD. Additionally, we conducted stratified analyses according to gender (male and female), age (< 65 years old, 65–70 years old, 70–75 years old, > 75 years old), hypertension (yes or no), diabetes (yes or no), and stroke (yes or no). The method of the U.K. Biobank cohort is similar to that of the Binhai cohort. All analyses were performed using R software (version 4.1.2). The *p-*values for all tests were two-sided, and *P* < 0.05 was considered significant.

## Result

### Baseline characteristics

The graphical abstract shows the overview of the study. During a median follow-up of 35 months, a total of 14,435 patients developed CKD among the 126,109 participants without CKD at baseline. The baseline characteristics of the participants according to CVAI quartile are shown in **Table 1**. Individuals in the highest quartile of CVAI had higher values of WHtR, VAI, ABSI, LAP, and BRI, as well as blood pressure and TG, while levels of HDL-c and eGFR were lower (*P* < 0.001). There were statistical differences observed in smoking status, drinking frequency, exercise frequency, and dietary habits across the groups. In the quantile groups of CVAI, the incidence rates over the 3-year follow-up were 2,478 (7.89%), 3,219 (10.21%), 3,899 (12.37%), and 4,830 (15.32%). Overall, the Cochran-Armitage trend test revealed a significant increase in the incidence (*P*-trend < 0.001). The baseline characteristics of the participants, stratified by gender and outcome, are shown in **Supplementary Tables S2**, **S3**.

**Table 1:** Baseline characteristics stratified by the quartile of CVAI (Binhai cohort).

Characteristics	1st	2nd	3rd	4th	*p* value
	<94.55 (n = 31521)	94.55-114.06 (n = 31541)	114.06-135.09 (n = 31517)	>135.09 (n = 31530)	
Male	18756 (59.5)	13977 (44.3)	11803 (37.4)	13367 (42.4)	<0.001^a^
Age, years	66.87 (6.37)	67.82 (6.09)	69.08 (6.29)	70.66 (6.62)	<0.001^a^
BMI, kg/m^2^	22.16 (2.09)	23.84 (1.86)	25.40 (2.04)	28.07 (2.59)	<0.001^a^
WC, cm	78.18 (4.99)	82.94 (4.63)	86.89 (5.05)	95.23 (6.92)	<0.001^a^
WHtR	0.47 (0.03)	0.50 (0.03)	0.53 (0.03)	0.58 (0.04)	<0.001^a^
VAI	1.18 (0.65)	1.75 (0.94)	2.22 (1.27)	2.68 (1.57)	<0.001^a^
ABSI	0.077 (0.005)	0.078 (0.005)	0.079 (0.005)	0.081 (0.005)	<0.001^a^
LAP	18.64 (9.20)	31.17 (12.66)	43.48 (18.03)	64.46 (29.09)	<0.001^a^
BRI	2.86 (0.52)	3.46 (0.52)	4.00 (0.62)	5.07 (0.94)	<0.001^a^
SBP, mmHg	125.32 (11.94)	127.32 (12.01)	129.43 (12.31)	132.30 (13.06)	<0.001^a^
DBP, mmHg	77.57 (7.17)	78.28 (7.05)	78.98 (9.75)	79.78 (7.46)	<0.001a
Laboratory tests					
FBG, mmol/L	5.43 (0.86)	5.58 (0.94)	5.70 (0.99)	5.90 (1.08)	<0.001^a^
HGB, g/L	140.59 (13.69)	140.47 (13.45)	140.76 (13.32)	142.46 (13.49)	<0.001^a^
TC, mmol/L	5.12 (0.98)	5.21 (1.02)	5.236 (1.03)	5.22 (1.05)	<0.001^a^
TG, mmol/L	1.18 (0.51)	1.46 (0.62)	1.69 (0.73)	1.90 (0.83)	<0.001^a^
HDL-C, mmol/L	1.57 (0.40)	1.42 (0.34)	1.36 (0.32)	1.29 (0.31)	<0.001^a^
LDL-C, mmol/L	2.72 (0.89)	2.85 (0.92)	2.91 (0.92)	2.91 (0.93)	<0.001^a^
TBIL, μmol/L	14.06 (5.12)	13.80 (4.99)	13.84 (4.98)	14.04 (5.09)	<0.001^a^
ALT, U/L	20.52 (14.89)	21.43 (14.73)	22.25 (23.57)	23.35 (17.50)	<0.001^a^
AST, U/L	22.60 (11.41)	22.17 (10.01)	22.20 (10.60)	22.56 (13.00)	<0.001^a^
Cr, mol/L	70.66 (14.52)	69.13 (14.43)	68.66 (14.49)	69.89 (14.71)	<0.001^a^
eGFR, ml/min/1.73m²	89.26 (14.23)	87.19 (13.75)	85.55 (13.42)	84.40 (13.45)	<0.001^a^
BUN, mg/dL	5.52 (1.37)	5.43 (1.32)	5.42 (1.31)	5.47 (1.33)	<0.001^a^
Smoking					
Current	8249 (26.17)	6470 (20.51)	5646 (17.91)	5715 (18.13)	<0.001^b^
Never	21001 (66.63)	22999 (72.91)	23669 (75.10)	22990 (72.91)	
Quit	2271 (7.20)	2072 (6.57)	2202 (6.99)	2825 (8.96)	
Drinking					
Never	22968 (72.87)	24928 (79.03)	25525 (80.99)	24530 (77.79)	<0.001^b^
1/week	4149 (13.16)	3448 (10.93)	3205 (10.17)	3755 (11.91)	
1-7/week	1224 (3.88)	960 (3.04)	846 (2.68)	967 (3.07)	
>7/week	3180 (10.09)	2205 (6.99)	1941 (6.16)	2278 (7.22)	
Exercise					
Never	8184 (25.96)	7517 (23.83)	7101 (22.53)	7566 (24.00)	<0.001^b^
1/week	1795 (5.69)	1965 (6.23)	1817 (5.76)	1809 (5.74)	
1-7/week	5154 (16.36)	5132 (16.27)	5474 (17.37)	5597 (17.75)	
>7/week	16388 (51.99)	16927 (53.67)	17125 (54.33)	16558 (52.51)	
Dietary conditions					
Balanced diet	29787 (94.50)	29923 (94.87)	29870 (94.77)	29681 (94.13)	<0.001^b^
Imalanced diet	1734 (5.50)	1618 (5.13)	1647 (5.23)	1849 (5.86)	
new cases of CKD	2487 (7.89)	3219 (10.21)	3899 (12.37)	4830 (15.32)	<0.001^b^

^a^Analysis of Variance, ^b^Chi-square test. Normally distributed data are expressed as mean and SDs, non-normally distributed data are expressed as median and quartiles, the rest are expressed as counts and percentages. SBP, systolic blood pressure; DBP, diastolic blood pressure; FBG, fasting blood glucose; HGB, haemoglobin; TC, total cholesterol; TG, triglyceride; HDL-c, high-density lipoprotein cholesterol; LDL-c, low-density lipoprotein cholesterol; TBIL, total bilirubin; AST, aspartate transaminase; ALT, alanine transaminase; Cr, creatinine; eGFR, estimated glomerular filtration rate; BUN, blood urea nitrogen.

In the U.K. Biobank cohort, during a median follow-up of 161 months, a total of 358,918 patients developed CKD among the 22,853 participants. The baseline characteristics of the participants according to CVAI quartile are shown in **Supplementary Tables S4**–**S6**.

### Correlation analyses of obesity indexes and estimated glomerular filtration rate

**Figure 2:**
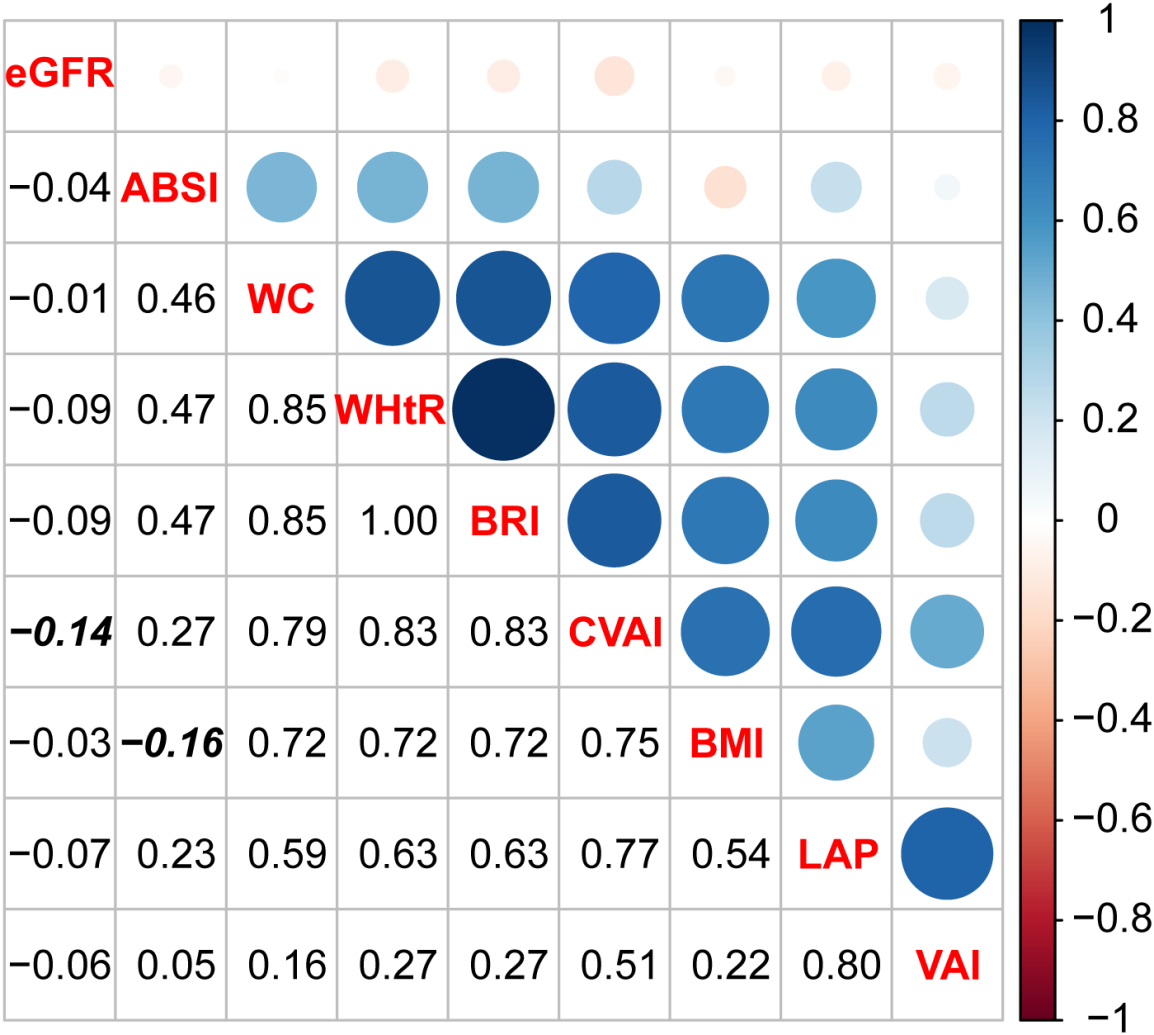
Spearman correlation analysis between obesity indexes and renal function.

In the Binhai cohort, eight obesity indexes were analyzed: ABSI, BMI, BRI, CVAI, LAP, VAI, WC, and WHtR. The results revealed positive correlations among all the obesity indexes, except for the negative correlation between BMI and ABSI (coefficient = -0.16). All eight obesity indexes were negatively correlated with eGFR, with CVAI exhibiting the most significant negative correlation (coefficient = -0.14). The details are presented in **Figure 2**. Similar results were obtained in the replication cohort, the UKB, as shown in **Supplementary Figure S1**.

### Relationship between baseline obesity indexes and risk of chronic kidney disease

We employed a Cox proportional hazards regression model to evaluate the efficiency of baseline obesity indexes in predicting the risk of CKD. The eight obesity indexes were categorized into quartiles, and three models were constructed. As shown in **Table 2**, in Model 3, CVAI, VAI, and LAP were significantly correlated with the risk of CKD incidence (*P* < 0.05). Specifically, for CVAI, with increasing quartile levels, the HR (95% CI) of Q2, Q3, and Q4 in comparison with Q1 were 1.10 (1.04-1.16), 1.14 (1.08-1.21) and 1.22 (1.16-1.30), respectively, demonstrating a significant upward trend (*P* for trend < 0.001). The risk of CKD in the Q4 group of VAI was 1.16 times higher than Q1, and the risk of CKD in the Q4 group of LAP was 1.26 times higher than Q1. The VAI and LAP were positively associated with the risk of CKD after adjusting for all confounders (*P* for trend < 0.001). Moreover, the BRI, WC, WHtR, and BMI were associated with an increased risk of CKD after adjusting for all confounders (*P* for trend < 0.001).

**Table 2:** The relationships between baseline obesity indexes and risk of chronic kidney disease (Binhai cohort).

Variable	N	Model 1	Model 2	Model 3
		HR (95%CI)	HR (95%CI)	HR (95%CI)
CVAI				
Q1: <94.55	31521	1.0 (ref)	1.0 (ref)	1.0 (ref)
Q2: 94.55-114.06	31541	1.29 (1.22-1.36)	1.17 (1.11-1.23)	1.10 (1.04-1.16)
Q3: 114.06-135.09	31517	1.55 (1.48-1.63)	1.28 (1.22-1.35)	1.14 (1.08-1.21)
Q4: >135.09	31530	1.95 (1.86-2.05)	1.47 (1.39-1.54)	1.22 (1.16-1.30)
*p* for trend		<0.001	<0.001	<0.001
VAI				
Q1: <1.10	31771	1.0 (ref)	1.0 (ref)	1.0 (ref)
Q2: 1.10-1.62	31040	1.17 (1.11-1.22)	1.14 (1.09-1.20)	1.09 (1.04-1.16)
Q3: 1.62-2.45	31838	1.24 (1.18-1.30)	1.20 (1.14-1.26)	1.13 (1.06-1.21)
Q4: >2.45	31460	1.30 (1.24-1.37)	1.25 (1.19-1.31)	1.16 (1.05-1.29)
*p* for trend		<0.001	<0.001	0.041
LAP				
Q1: <21.84	31472	1.0 (ref)	1.0 (ref)	1.0 (ref)
Q2: 21.84-33.20	31593	1.18 (1.12-1.24)	1.18 (1.12-1.24)	1.11 (1.05-1.16)
Q3: 33.20-50.40	31586	1.23 (1.17-1.29)	1.23 (1.17-1.29)	1.12 (1.06-1.18)
Q4: >50.40	31458	1.42 (1.36-1.49)	1.42 (1.36-1.49)	1.26 (1.18-1.35)
*p* for trend		<0.001	<0.001	<0.001
BRI				
Q1: <3.13	32256	1.0 (ref)	1.0 (ref)	1.0 (ref)
Q2: 3.13-3.70	31456	1.06 (1.01-1.11)	1.04 (0.99-1.09)	0.98 (0.93-1.03)
Q3: 3.70-4.43	30574	1.25 (1.19-1.31)	1.18 (1.13-1.24)	1.06 (1.01-1.12)
Q4: >4.43	31823	1.48 (1.41-1.55)	1.30 (1.24-1.36)	1.12 (1.06-1.17)
*p* for trend		<0.001	<0.001	<0.001
WC				
Q1: <80.00	26817	1.0 (ref)	1.0 (ref)	1.0 (ref)
Q2: 80.00-85.00	38511	0.97 (0.92-1.02)	1.05 (1.00-1.11)	1.00 (0.96-1.05)
Q3: 85.00-90.00	30369	1.07 (1.02-1.12)	1.18 (1.12-1.24)	1.06 (1.01-1.12)
Q4: >90.00	30412	1.20 (1.15-1.26)	1.34 (1.27-1.40)	1.13 (1.08-1.19)
*p* for trend		<0.001	<0.001	<0.001
ABSI				
Q1: <0.076	35563	1.0 (ref)	1.0 (ref)	1.0 (ref)
Q2: 0.076-0.079	32341	1.05 (1.00-1.09)	1.02 (0.97-1.06)	1.00 (0.96-1.05)
Q3: 0.079-0.082	27999	1.05 (1.00-1.10)	0.99 (0.95-1.04)	0.97 (0.93-1.02)
Q4: >0.082	30206	1.22 (1.17-1.28)	1.05 (1.00-1.10)	1.05 (1.00-1.10)
*p* for trend		<0.001	0.055	0.061
WHtR				
Q1: <0.49	34762	1.0 (ref)	1.0 (ref)	1.0 (ref)
Q2: 0.49-0.52	31060	1.06 (1.01-1.12)	1.03 (0.98-1.08)	0.97 (0.93-1.02)
Q3: 0.52-0.55	26034	1.26 (1.20-1.33)	1.19 (1.13-1.25)	1.08 (1.02-1.13)
Q4: >0.55	34253	1.46 (1.40-1.53)	1.29 (1.23-1.35)	1.11 (1.06-1.16)
*p* for trend		<0.001	<0.001	<0.001
BMI				
Q1: <22.86	30686	1.0(ref)	1.0 (ref)	1.0 (ref)
Q2: 22.86-24.57	32609	1.00 (0.96-1.05)	1.07 (1.02-1.13)	1.00 (0.95-1.05)
Q3: 24.57-26.75	31364	1.15 (1.10-1.21)	1.24 (1.18-1.30)	1.10 (1.05-1.15)
Q4: >26.75	31450	1.26 (1.20-1.32)	1.36 (1.30-1.43)	1.14 (1.09-1.20)
*p* for trend		<0.001	<0.001	<0.001

These findings were consistent with the results of substitution analysis in the U.K. Biobank cohort. According to the CVAI quartile levels, the HR (95% CI) of Q2, Q3, Q4 in comparison with Q1 were 1.22 (1.16-1.28), 1.33 (1.26-1.28) and 1.63 (1.54-1.73). The details are shown in **Table 3**.

**Table 3:** The relationships between baseline obesity indexes and risk of chronic kidney disease (U.K. Biobank cohort).

Variable	N	Model 1	Model 2	Model 3
		HR (95%CI)	HR (95%CI)	HR (95%CI)
CVAI				
Q1:<85.94	89723	1.0 (ref)	1.0 (ref)	1.0 (ref)
Q2:85.94-118.78	89727	1.87 (1.78-1.96)	1.39 (1.32-1.46)	1.22 (1.16-1.28)
Q3:118.78-153.63	89746	2.62 (2.50-2.74)	1.80 (1.71-1.89)	1.33 (1.26-1.28)
Q4:>153.63	89722	4.26 (4.07-4.45)	2.80 (2.67-2.94)	1.63 (1.54-1.73)
*P* for trend		<0.001	<0.001	<0.001
VAI				
Q1:<1.04	89011	1.0 (ref)	1.0 (ref)	1.0 (ref)
Q2:1.04-1.66	90776	1.31 (1.25-1.37)	1.20 (1.15-1.26)	1.01 (0.97-1.06)
Q3:1.66-2.67	89280	1.69 (1.62-1.76)	1.48 (1.42-1.54)	1.08 (1.02-1.14)
Q4:>2.67	89851	2.21 (2.13-2.30)	1.94 (1.87-2.02)	1.15 (1.07-1.23)
*P* for trend		<0.001	<0.001	<0.001
LAP				
Q1:<22.89	89732	1.0 (ref)	1.0 (ref)	1.0 (ref)
Q2:22.89-41.54	89752	1.61 (1.54-1.68)	1.34 (1.28-1.41)	1.18 (1.13-1.24)
Q3:41.54-71.55	89696	2.11 (2.02-2.20)	1.66 (1.59-1.73)	1.29 (1.22-1.35)
Q4:>71.55	89738	2.89 (2.77-3.01)	2.30 (2.21-2.40)	1.53 (1.45-1.63)
*P* for trend		<0.001	<0.001	<0.001
BRI				
Q1:<3.03	89379	1.0 (ref)	1.0 (ref)	1.0 (ref)
Q2:3.03-3.93	90289	1.51 (1.44-1.58)	1.29 (1.23-1.35)	1.13 (1.07-1.18)
Q3:3.93-4.97	89419	2.04 (1.96-2.14)	1.61 (1.53-1.68)	1.24 (1.18-1.30)
Q4:>4.97	89831	3.24 (3.11-3.38)	2.47 (2.37-2.58)	1.53 (1.46-1.61)
*P* for trend		<0.001	<0.001	<0.001
WC				
Q1:<80.00	82422	1.0 (ref)	1.0 (ref)	1.0 (ref)
Q2:80.00-90.00	105509	1.54 (1.47-1.61)	1.41 (1.34-1.47)	1.18 (1.12-1.23)
Q3:90.00-99.00	87425	2.05 (1.96-2.14)	1.82 (1.74-1.91)	1.30 (1.23-1.37)
Q4:>99.00	83562	3.14 (3.00-3.27)	2.74 (2.62-2.87)	1.58 (1.50-1.66)
*P* for trend		<0.001	<0.001	<0.001
ABSI				
Q1:<0.073	94235	1.0 (ref)	1.0 (ref)	1.0 (ref)
Q2:0.073-0.077	89676	1.34 (1.29-1.40)	1.21 (1.16-1.26)	1.11 (1.06-1.16)
Q3:0.077-0.081	95626	1.65 (1.59-1.72)	1.35 (1.29-1.41)	1.17 (1.12-1.22)
Q4:>0.081	79381	2.23 (2.15-2.32)	1.59 (1.52-1.66)	1.31 (1.25-1.38)
*P* for trend		<0.001	<0.001	<0.001
WHtR				
Q1:<0.48	85543	1.0 (ref)	1.0 (ref)	1.0 (ref)
Q2:0.48-0.53	94782	1.52 (1.45-1.59)	1.30 (1.24-1.36)	1.13 (1.08-1.19)
Q3:0.53-0.58	89695	2.08 (1.99-2.18)	1.63 (1.55-1.70)	1.25 (1.19-1.31)
Q4:>0.58	88898	3.29 (3.15-3.43)	2.49 (2.39-2.60)	1.54 (1.47-1.62)
*P* for trend		<0.001	<0.001	<0.001
BMI				
Q1:<24.10	89721	1.0 (ref)	1.0 (ref)	1.0 (ref)
Q2:24.10-26.68	89666	1.37 (1.31-1.43)	1.22 (1.16-1.27)	1.02 (0.98-1.07)
Q3:26.68-29.81	89832	1.77 (1.70-1.85)	1.52 (1.45-1.58)	1.10 (1.05-1.15)
Q4:>29.81	89699	2.62 (2.51-2.72)	2.34 (2.25-2.43)	1.37 (1.31-1.43)
*P* for trend		<0.001	<0.001	<0.001
Hips				
Q1:<97.00	76640	1.0 (ref)	1.0 (ref)	1.0 (ref)
Q2:97.00-102.00	107509	1.25 (1.20-1.30)	1.15 (1.10-1.20)	1.01 (0.97-1.05)
Q3:102.00-108.00	92802	1.49 (1.42-1.55)	1.35 (1.29-1.40)	1.06 (1.02-1.11)
Q4:>108.00	81967	2.02 (1.94-2.10)	1.94 (1.87-2.02)	1.26 (1.21-1.32)
*P* for trend		<0.001	<0.001	<0.001
WHtR				
Q1:<0.80	84787	1.0 (ref)	1.0 (ref)	1.0 (ref)
Q2:0.80-0.87	91393	1.48 (1.41-1.55)	1.39 (1.33-1.46)	1.18 (1.13-1.24)
Q3:0.87-0.94	98544	1.85 (1.77-1.93)	1.82 (1.73-1.91)	1.31 (1.25-1.38)
Q4:>0.94	84194	2.78 (2.67-2.90)	2.62 (2.49-2.76)	1.54 (1.45-1.62)
*P* for trend		<0.001	<0.001	<0.001
BAI				
Q1:<25.63	89821	1.0 (ref)	1.0 (ref)	1.0 (ref)
Q2:25.63-28.50	89103	1.15 (1.11-1.20)	1.21 (1.16-1.25)	1.03 (0.99-1.07)
Q3:28.50-32.16	90197	1.23 (1.19-1.28)	1.46 (1.40-1.52)	1.12 (1.07-1.17)
Q4:>32.16	89797	1.59 (1.54-1.65)	2.15 (2.06-2.25)	1.36 (1.30-1.42)
*P* for trend		<0.001	<0.001	<0.001

### The dose-response relationships between obesity indexes and risk of CKD

**Figure 3:**
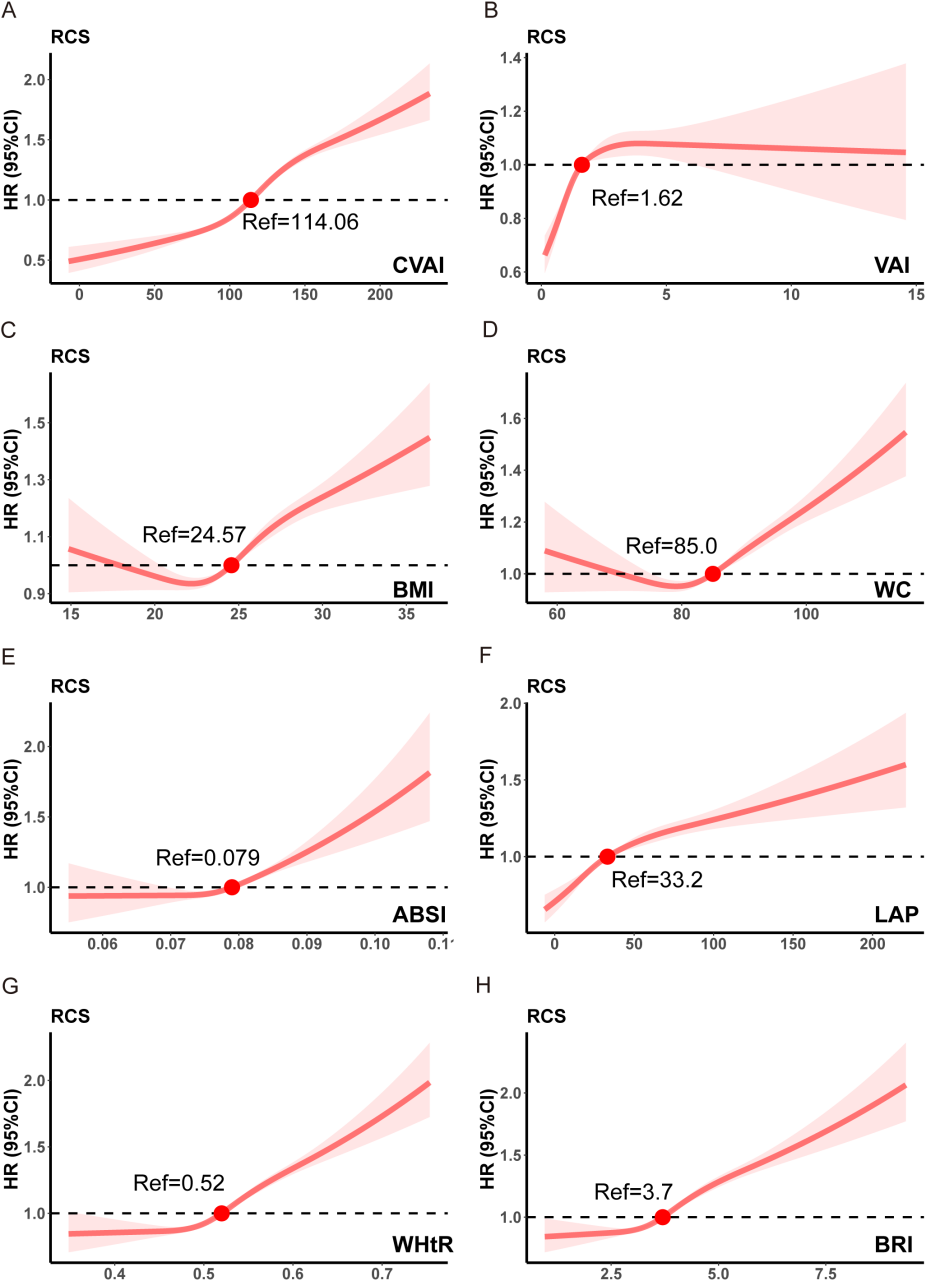
The dose-response relationships between obesity indexes and risk of CKD. **(A)** CVAI, Chinese visceral adiposity index. **(B)** VAI, visceral adiposity index. **(C)** BMI, body mass index. **(D)** WC, waist circumference. **(E)** ABSI, a body shape index. **(F)** LAP, lipid accumulation product. **(G)** WHtR, waist-height ratio. **(H)** BRI, body roundness index. Analyses were adjusted for gender, age, SBP, DBP, LDL-c, HDL-c, TC, TG, eGFR at baseline, smoking, drinking, exercise, diet, diabetes, hypertension and stroke.

As shown in **Figure 3**, all obesity indexes, including CVAI, VAI, WHtR, BRI, LAP, ABSI, BMI, and WC, exhibited non-linear dose-response relationships with the risk of CKD (*P* for non-linear < 0.001). We observed a J-shaped dose-response relation between CVAI, WHtR, ABSI, BRI, LAP, and the risk of CKD, with an increased risk as these obesity indexes elevated. Specifically, the risk of CKD was significantly higher when CVAI > 114.06, WHtR > 0.52, ABSI > 0.079, BRI > 3.7, and LAP > 33.2. In addition, BMI and WC demonstrated a U-shaped relationship with CKD risk, with inflection points observed at 24.57 kg/m^2^ for BMI and 85 cm for WC. However, VAI exhibited an inverted U-shaped relationship with CKD risk, where the risk sharply increased when VAI > 1.62, but then decreased as VAI > 4.

Findings from the UKB were similar (**Supplementary Figure S2**). CVAI, VAI, WHtR, BRI, LAP, ABSI, BMI, WC, Hips, and BAI, exhibited non-linear dose-response relationships with the risk of CKD (*P* for non-linear < 0.001).

### Sensitivity and specificity analysis

We adopted the ROC curve to evaluate the sensitivity and specificity of obesity indexes as prognostic indicators for CKD. Compared to the other 9 obesity indexes, CVAI demonstrated the highest predictive ability for both men and women, with AUC values of 0.564 (0.556-0.571) and 0.588 (0.581-0.594), respectively, in Binhai cohort. In addition, the top three obesity indexes for both genders were CVAI, WHtR, and BRI.

The details are shown in **Supplementary Figure S3**. Equivalent results were obtained from the U.K. Biobank cohort, as illustrated in **Supplementary Figure S4**.

### Subgroup analyses

**Figure 4:**
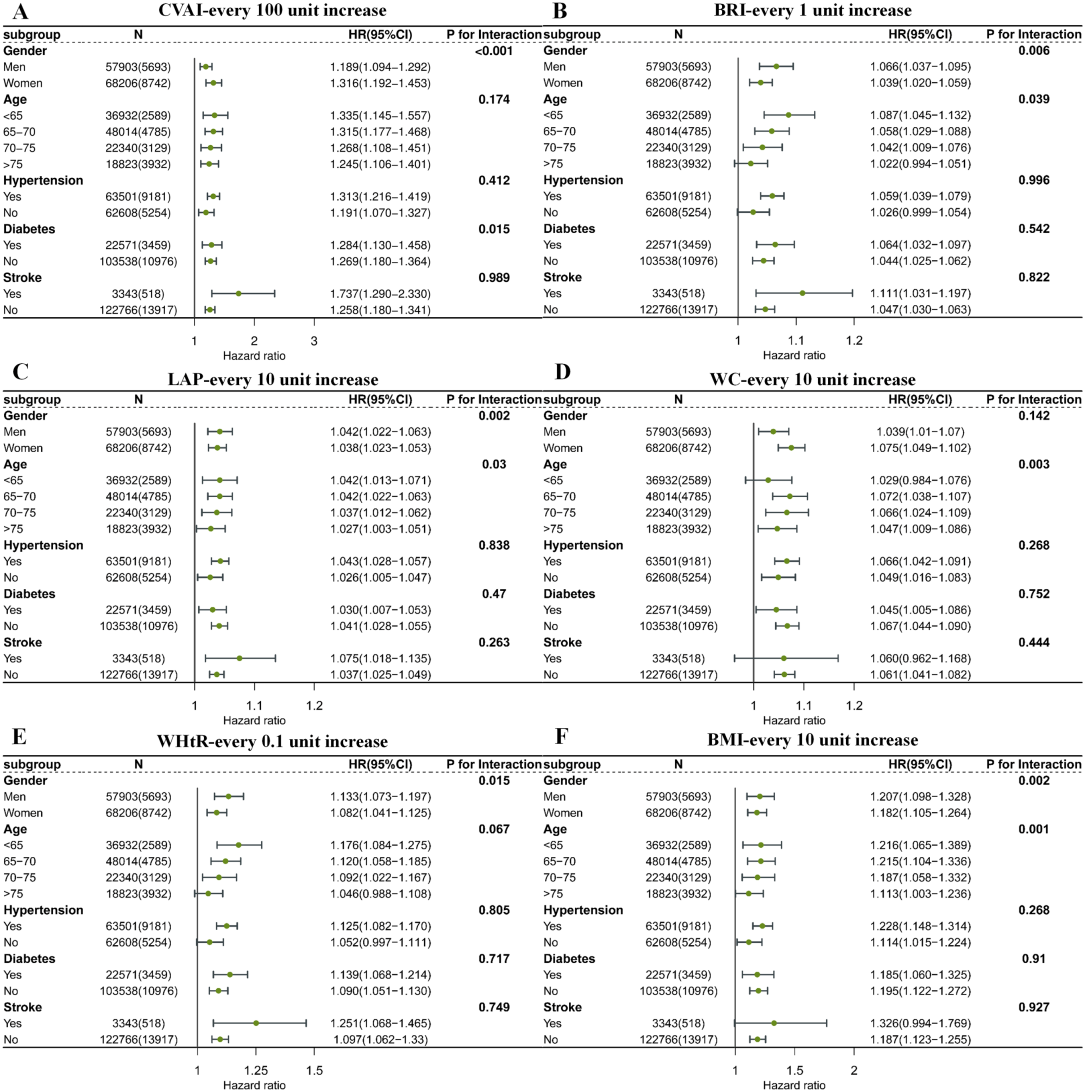
The stratified analyses of the associations between obesity indexes and risk of CKD. **(A)** CVAI, Chinese visceral adiposity index. **(B)** BRI, body roundness index. **(C)** LAP, lipid accumulation product. **(D)** WC, waist circumference. **(E)** WHtR, waist-height ratio. **(F)** BMI, body mass index. Analyses were adjusted for gender, age, SBP, DBP, LDL-c, HDL-c, TC, TG, smoking, drinking, exercise, diet, diabetes, hypertension and stroke.

As shown in **Figure 4**, in the subgroup analyses stratified by sex, the results showed that for every 100-unit increase in CVAI, the risk of CKD in women significantly increased by 36.1% (HR: 1.361; 95% CI, 1.192-1.453, [*P* <0.001]), which was significantly higher than in men (HR: 1.189; 95% CI, 1.094-1.292, [*P* <0.001]). In the subgroup analysis stratified by diabetes, individuals with diabetes had a significantly higher risk of CKD (HR: 1.284; 95% CI, 1.130-1.458, *P* = 0.015) compared to those without diabetes. Furthermore, younger individuals, particularly men, were more likely to be affected by LAP and develop CKD (*P* for interaction < 0.05). Similarly, younger males were more susceptible to the impact of BMI and BRI and developed CKD (*P* for interaction < 0.05). For WC, the highest risk of CKD was observed in individuals aged 65–70 years (*P* for interaction = 0.003). In terms of WHtR, the risk of CKD in males was significantly higher than in females (*P* for interaction = 0.015). Furthermore, considering the ethnic diversity of the European population, we assessed the predictive value of CVAI for incident CKD across different ethnic subgroups. In our study, White participants accounted for 94.7% of the UK Biobank cohort (**Supplementary Table S9**), which is consistent with previous reports showing proportions ranging from 94.2% to 95.8% (20–22). The results demonstrated that CVAI maintained a stable predictive value for CKD in the White, Black, and Asian subgroups under both Model 1 and Model 2 adjustments (**Supplementary Table S10**). Moreover, considering the difference in median follow-up time between the two cohorts, we conducted a subgroup analysis in the U.K. Biobank cohort with a 35-month follow-up period. The results showed that the HR for CKD per every 100-unit increase in CAVI was 2.69 (95% CI: 2.50–2.89) in Model 1 (**Supplementary Table S11**). This finding further supports the positive association between elevated CAVI and increased CKD risk in the European population within a comparable follow-up duration.

After adjustment, no significant differences between subgroups were observed in VAI and ABSI (**Supplementary Tables S7**, **S8**). The results from the U.K. Biobank cohort are consistent with these findings, and the details are shown in **Supplementary Figure S5**.

## Discussion

This study assessed the association between obesity indexes and the risk of CKD using data from the Binhai and U.K. Biobank cohorts. To our knowledge, this is the first prospective double-cohort study to examine the critical role of CVAI in CKD risk on such a large scale. Our findings demonstrate that CVAI, a novel index of visceral adiposity, exhibits strong associations with CKD risk, exceeding established indicators such as BMI and WC, and is significantly correlated with visceral fat burden, as defined by CT, as well as several key renal risk factors. This study provides new insights into the role of adiposity in CKD pathogenesis and demonstrates the importance of using advanced obesity indexes for risk stratification in clinical settings.

It is worth noting that the subgroup analysis of CVAI showed that females and individuals with diabetes exhibited a higher susceptibility to CKD development associated with elevated CVAI levels. In line with previous reports, in 2017, it was estimated that in the approximately 700 million people with CKD globally, females had a prevalence of 1.29 times higher than males (1). Notably, gender-specific differences in CKD prevalence vary by country, with some regions reporting prevalence in females as twice that in males. This may be due to women’s longer life expectancy and the use of equations to eGFR, which could lead to the overdiagnosis of CKD in women (23). Previous studies have reported that CVAI appears to be more valuable than other obesity indexes in predicting renal impairment in females (24), which is consistent with the findings of our study. In contrast to males, females exhibit distinct patterns of adipose tissue distribution, characterized by greater fat accumulation in the subcutaneous depot prior to menopause, followed by a shift toward increased visceral fat deposition and accrual after menopause (25). This shift is accompanied by a corresponding rise in CKD risk. Research in animal models demonstrates that estrogen signaling deficiency promotes metabolic dysregulation characterized by adiposity redistribution toward visceral depots and disturbances in glucose metabolism and insulin sensitivity (26). Furthermore, insulin resistance induced glucolipotoxicity manifests in renal parenchyma through altered podocyte viability and tubular function, mechanistically contributing to progressive renal dysfunction (27). Taken together, this may explain in part why CVAI demonstrates a stronger association with CKD risk in females, while the underlying mechanisms require further investigation. Although the prevalence of CKD is higher in females, the progression of kidney disease generally is reported to be more rapid in males (28), as evidenced by large cohort studies demonstrating increased risks of renal replacement therapy among males (29). A large systematic review and meta-analysis found that the association between obesity and CKD varies by gender, with obesity increasing the risk of CKD for females more than for males (30). Similarly, consistent with our results, the gender subgroup analysis revealed that WHtR and BRI exhibited a higher risk in males compared to females, unlike CVAI. We propose that WHtR and BRI are relatively simple indexes that cannot be corrected by gender, age, BMI, and HDL-c. Additionally, we identified a J-shaped dose-response relation between CVAI, WHtR, BRI, and the risk of CKD, with inflection point values (CVAI: 114.06, WHtR: 0.52, BRI: 3.7). These nonlinear transition points indicate critical biological thresholds where metabolic disorders may accelerate renal function decline, enabling focused treatment surveillance for individuals exceeding these cutoffs.

Diabetes, recognized as the leading cause of CKD globally, is strongly associated with obesity (31). Adiposity contributes to insulin resistance and chronic low-grade inflammation, both of which exacerbate renal dysfunction (32, 33). Similar results were also observed in our studies that the risk of CKD affected by the abdominal adiposity index CVAI was different in patients with diabetes and those without diabetes. An accumulating body of studies has investigated the association between obesity indexes and CKD risk, but the conclusions remain inconsistent. A large prospective cohort study in Sweden demonstrated significant associations between anthropometric measures, including BMI, WC, WHtR, and body fat percentage, and the incidence of CKD (34). However, other studies have reported that WHtR, rather than BMI, is more closely associated with both the incidence and mortality of CKD (35). According to some researchers, ABSI may serve as a better indicator of CKD risk than BMI (36). Moreover, the body adiposity index has been proposed as a potential predictive tool for identifying obesity-related CKD in the early stage (37). Although fat distribution patterns in Asians differ from those in Europeans and Americans, our findings demonstrate that CVAI has the most predictive value for CKD progression among all obesity indexes. Specifically, results from the Binhai and U.K. Biobank cohorts indicate that CVAI, WHtR, and BRI consistently rank as the top three obesity indexes in both males and females, and CVAI shows the highest average predictive AUC values of ROC analysis. In-depth comparison of the parameter composition of various obesity indexes, together with the exploration of potential biological mechanisms underlying these parameters, may reveal why individuals with greater CVAI in our study exhibited a higher risk of renal impairment. Compared to the other 9 obesity indexes, CVAI incorporates multiple parameters, including BMI, WC, TG, and HDL-c, reflecting increased ectopic fat depots and providing a more comprehensive depiction of abnormal lipid metabolism. The visceral adipose tissue is the main contributor to systemic inflammation in obesity, as it can produce and secrete a greater quantity of pro-inflammatory cytokines, such as leptin, adiponectin, interleukin 6, and tumor necrosis factor-α, which is compared to subcutaneous fat (38–40). These cytokines and adipokines induce oxidative stress, inflammation, and even trigger fibrotic changes in the kidney, ultimately leading to renal damage (38). Hence, this suggests that obesity indexes incorporating lipid metabolic parameters may provide a more comprehensive understanding of the contribution of lipotoxicity to the progression of kidney disease.

Currently, research available on CVAI remains relatively limited, with existing studies exhibiting considerable heterogeneity in the final enrolled cohort sample size, assessment of outcome, study type, and statistical analyses employed. A longitudinal survey demonstrated that six insulin resistance indexes were associated with rapid kidney function decline (RKFD) in Chinese with normal renal function over age 45, while CVAI was the best index for predicting further progression to CKD (41). A retrospective cross-sectional study indicated that the CVAI is significantly and negatively correlated with eGFR, particularly showing superior screening efficiency in the female population (24). A cross-sectional survey revealed that the CVAI are significantly and positively associated with CKD prevalence in a Korean population (42). A previous study in the Beijing Health Examination cohort, consisting of 23,522 participants aged 20 to 80 years, showed a significant positive correlation between CVAI and carotid plaque risk, characterized by a non-linear dose-response relationship, with a stronger association observed in men compared to women (43). Other studies have indicated that CVAI may be a reliable index to identify high-risk groups of atherosclerotic cardiovascular disease (44). A single-center observational cohort study demonstrated that insulin resistance (IR) surrogate indicators, such as CVAI, offer advantages of simplicity, cost-effectiveness, and insulin independence, highlighting their significant potential as valuable clinical indicators for reflecting IR levels (45).

The current study has several strengths, this large-scale, community-based study, is the first to address the limitations of previous research that focused on a single ethnic group and lacked Hips metrics. It provides robust evidence for the positive correlation between CVAI and the incidence of CKD across different ethnicities, confirming the value of CVAI as an effective obesity index for predicting CKD risk. In addition, in subgroup analysis, we found that the association between CVAI and progression to CKD was more significant in subjects in the female subgroup or with diabetes. There are limitations that should be considered in our study. The albumin-to-creatinine ratio, an important marker for assessing renal function and detecting kidney injury, was not included in the current analysis.

## Conclusion

In conclusion, in these two large prospective cohort studies, we found that obesity indexes CVAI, WHtR, ABSI, BRI, and LAP are significantly positively correlated with the risk of CKD. This underlines the critical role of these obesity indexes in identifying individuals at heightened risk for CKD, particularly in the continuing rise in the global obesity epidemic. Notably, among these obesity indexes, CVAI stands out as a simple, cost-effective indicator, and our findings indicate its applicability across both the Chinese population and other ethnic groups. These insights are crucial for guiding principles in early interventions, which may ultimately help reduce the burden of CKD, especially in high-risk groups.

## Data Availability

The raw data supporting the conclusions of this article will be made available by the authors, without undue reservation.
